# Improving After-Hours Echocardiography Communication: A Resident-Led Quality Improvement Project

**DOI:** 10.7759/cureus.108096

**Published:** 2026-05-01

**Authors:** Riya Kalra, Moeez Hassan, Muhammad Waqas, Clinton Cochran, Anuradha Prabhu

**Affiliations:** 1 Pediatrics, MetroHealth Medical Center, Cleveland, USA; 2 Pediatric Cardiology, University of South Dakota Sanford School of Medicine, Sioux Falls, USA; 3 Pediatrics, Essentia Health, Fargo, USA; 4 Pediatric Cardiology, University of Michigan, Ann Arbor, USA; 5 Pediatric Cardiology, Corewell Health William Beaumont School of Medicine, Royal Oak, USA

**Keywords:** after-hours, quality improvement projects, resident-led, systems level intervention, transthoracic echocardiogram

## Abstract

Background

In our paediatric inpatient units, after-hours echocardiogram orders frequently lacked proper communication between ordering providers and interpreting paediatric cardiologists. This gap contributed to imaging delays, inefficiencies, and potential patient safety risks. We aimed to improve provider notification compliance for after-hours echocardiogram orders.

Design

We designed a resident-led quality improvement (QI) project using sequential Plan-Do-Study-Act (PDSA) cycles. Baseline data were obtained from self-reported provider surveys. Compliance with communication was measured as the percentage of providers who self-reported “Always” notifying the on-call cardiologist at the time of order placement. Interventions included email reminders, poster reminders in work areas, and an electronic health record (EHR) modification requiring mandatory entry of indication and notification details before order completion. Three PDSA cycles were implemented over a 16-month period. Each cycle was monitored for compliance trends, with iterative adjustments based on feedback from frontline providers.

Results

Baseline compliance was low, with surveys highlighting frequent lapses in provider communication. The first PDSA cycle, involving email reminders, produced a trivial improvement in compliance, while the second cycle, involving posters, further reinforced the process. The most significant and sustained improvement followed the EHR modification in the third cycle, after which communication compliance exceeded 70% and was sustained over six months.

Conclusions

This resident-led project demonstrated that while simple, low-cost interventions produced modest gains, sustained improvement required an EHR-based solution, highlighting the value of combining educational and systems-level strategies to enhance patient safety.

## Introduction

Diagnostic echocardiography is essential for the timely evaluation and management of acutely ill pediatric patients. “After-hours orders,” defined in our institution as echocardiogram orders placed on weekends and after 4:00 PM on weekdays, often involve urgent or emergent cases, where miscommunication or missed communication can delay vital care [1,2]. Prior assessments at our institution revealed frequent gaps in communication between ordering providers, pediatric cardiologists, and pediatric cardiac sonographers. This presented both workflow inefficiencies and potential patient safety risks. We designed and implemented a quality improvement (QI) project to address these challenges using sequential Plan-Do-Study-Act (PDSA) cycles. The aim was to improve the quality and completeness of echocardiogram orders and increase ordering provider-to-interpreting provider communication from <30% to greater than 70%, with a primary outcome of provider communication compliance, defined as the proportion of ordering providers who self-reported “Always” notifying the on-call pediatric cardiologist at the time of order placement.

## Materials and methods

Context

This QI initiative was conducted in a tertiary-care pediatric unit, with approximately 70 beds, within a large academic medical center serving a diverse population of medical and surgical patients and functioning as a major training site for residents and nurse practitioners. After-hours echocardiogram orders were placed by residents, nurse practitioners, or pediatric hospitalists, with pediatric cardiologists and pediatric cardiac sonographers on call. Based on provider experience, it was evident that the process of echocardiogram ordering and notification of the on-call cardiologist needed structured improvement to ensure compliance. The QI team consisted of four pediatric residents at different levels of training and three pediatric cardiologists. Responses from all participating pediatric residents, combined internal medicine-pediatric residents, pediatric nurse practitioners, and pediatric hospitalists, collectively referred to as “ordering providers,” were included in the project.

Interventions

Three sequential PDSA cycles were implemented:

PDSA Cycle 1 (September 2020-November 2020)

Email reminders were sent to all participants regarding notification responsibilities. The initial email introduced the project, outlined the problem, and described the planned first cycle. Verbal awareness sessions supplemented this communication. Thereafter, weekly follow-up emails and verbal reminders were provided until the end of the cycle.

PDSA Cycle 2 (December 2020-July 2021)

**Figure 1:**
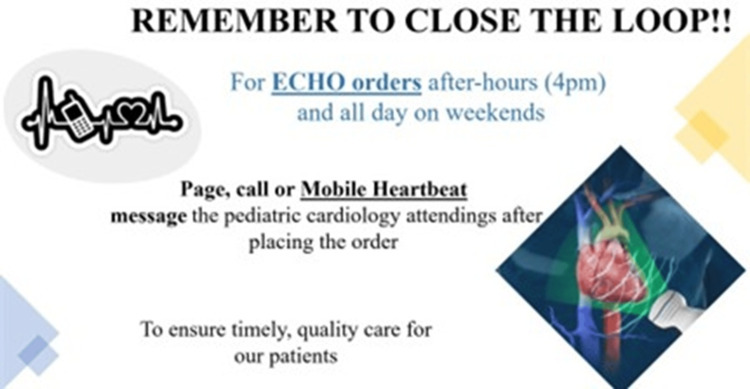
Educational poster displayed in resident, nurse practitioner, and hospitalist workrooms during PDSA Cycle 2, reinforcing notification responsibilities and communication steps. Mobile Heartbeat is the phone paging system used at the institution. PDSA: Plan-Do-Study-Act.

Educational posters (Figure 1) were displayed in resident, nurse practitioner, and hospitalist workrooms to reinforce communication steps. This intervention was supported by monthly follow-up emails and verbal reminders throughout the cycle.

In both of the above PDSA cycles, verbal reminders consisted of brief, structured discussions conducted during routine team huddles every 4 weeks.

PDSA Cycle 3 (August 2021-December 2021)

**Figure 2:**
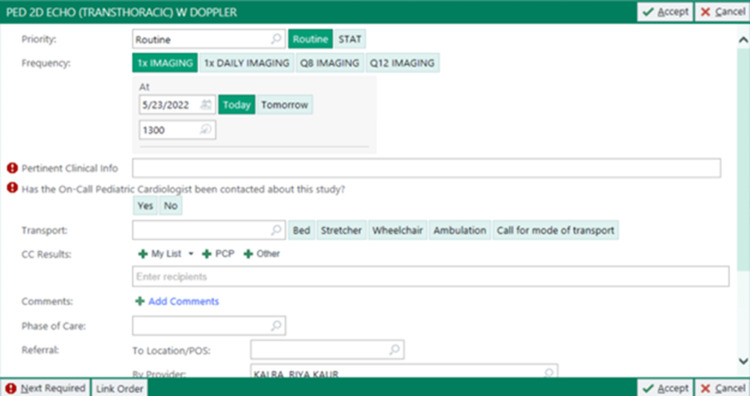
Electronic health record (EHR) modification implemented during PDSA Cycle 3, requiring mandatory documentation of indication and notification prior to order completion. Red markers indicate mandatory “hard stops” before placing an order. The order cannot be signed unless these fields are completed. PDSA: Plan-Do-Study-Act.

An electronic health record (EHR) modification was implemented, requiring mandatory documentation of indication and notification prior to order completion (Figure 2). In this context, “notification” refers to paging the pediatric cardiologist during or immediately after order placement to ensure timely review and completion of required documentation elements. This step functioned as an additional accountability and communication measure to support order completeness.

Study of the interventions

Compliance was tracked through self-reported provider surveys. Each PDSA cycle was informed by real-time feedback from frontline users who communicated any emerging workflow challenges. Early cycles focused on improving awareness and standardizing expectations, while subsequent iterations addressed system-level barriers.

Real-time feedback was collected through both structured and informal mechanisms. A survey distributed to participants included a dedicated field for reporting challenges, barriers, and suggestions, allowing for documented, contemporaneous feedback. In addition, members of the QI team were readily accessible via phone and text, enabling clinicians to communicate issues as they arose in real time. These interactions were primarily informal and were used to promptly identify and address workflow challenges during implementation.

The survey instrument was internally developed for this QI initiative. Survey responses included the categories “Always,” “Sometimes,” and “Never” with respect to provider notification. “Always” was defined as completion in 100% of applicable encounters, “Sometimes” as completion in more than 0% but less than 100% of applicable encounters, and “Never” as no completion in any applicable encounter. The instrument was not formally validated but was reviewed internally for clarity prior to use.

Measures

The primary outcome measure was self-reported communication compliance, defined as “Always” notifying the on-call cardiologist at the time of order placement. Secondary measures included process measures, such as completeness of the echocardiography order and adequacy of information provided, and balancing measures, such as provider-reported barriers and satisfaction.

The target of achieving ≥70% compliance with after-hours communication was defined a priori by the project team as a pragmatic benchmark for meaningful improvement. Given baseline adherence of fewer than 30%, this threshold was selected through multidisciplinary consensus as a realistic yet clinically significant goal within the constraints of existing workflow and communication systems.

A structured questionnaire sent to ordering providers was used to measure the completeness of the echocardiography order and communication with the on-call cardiologists. The questionnaire was divided into three key domains: (1) completeness of documentation, (2) adequacy of information provided, and (3) cardiologist notification. These measures were chosen to assess both the process and the outcome. To enhance response rates and minimize non-response bias, the survey was distributed twice during each PDSA cycle: once midway through the cycle and once at its conclusion. Surveys were administered prospectively and at consistent time points following each intervention phase to minimize recall bias and ensure comparability across cycles. Questionnaires were distributed electronically via institutional email, with reminder messages sent to maximize participation. The same survey instrument was used throughout the study period, with identical wording and response options for each cycle. Respondents were asked to reflect on their current practice within the current cycle rather than on distant experiences. Participation was voluntary, and responses were anonymized to encourage candid reporting. A single designated investigator collected all data to maintain consistency and reduce measurement bias.

While self-reported data are subject to response and social desirability bias, this approach allowed real-time assessment of provider perceptions during iterative QI cycles. These data were complemented by qualitative feedback and direct review of order completeness by pediatric cardiologists.

To establish that observed improvements were due to the intervention, no other significant changes in departmental policy or workflow occurred during the project period. Comparisons were made between preintervention and postintervention results to attribute changes directly to the interventions studied. Additionally, the durability of improvements was assessed in the 6 months following Cycle 3.

Analysis

Data were summarized as percentages and displayed in run charts across PDSA cycles. Run charts were interpreted using standard rules for detecting non-random variation.

Qualitative feedback from survey responses and real-time communications was analyzed using a thematic approach. Responses were reviewed to identify recurring barriers and facilitators, and resolutions were developed iteratively based on emerging patterns in the data. Given the QI nature of the project, the analysis was conducted by members of the study team, with themes refined through group discussion.

Resources and cost considerations

This QI initiative was implemented using existing institutional resources and did not require additional external funding. The primary inputs included staff time for meetings, educational outreach, and collaboration with health information technology personnel to design and implement the EHR modification. The EHR intervention leveraged existing system functionality and therefore incurred no direct financial cost related to software acquisition, licensing, or hardware. Informatics support was provided within standard departmental operations, and no additional equipment or infrastructure was required. These activities were integrated into routine operational and educational workflows and did not necessitate dedicated project staffing.

## Results

A total of 24 pediatric residents, 12 combined internal medicine-pediatric residents, six pediatric nurse practitioners, and six pediatric hospitalists participated in the project. Baseline surveys (36/48 responses, 75%) revealed that fewer than 30% of after-hours echocardiography orders were “Always” communicated appropriately to the on-call pediatric cardiology attending, confirming a substantial gap in communication practices and highlighting the need for intervention.

Cycle 1 (September-November 2020)

**Figure 3:**
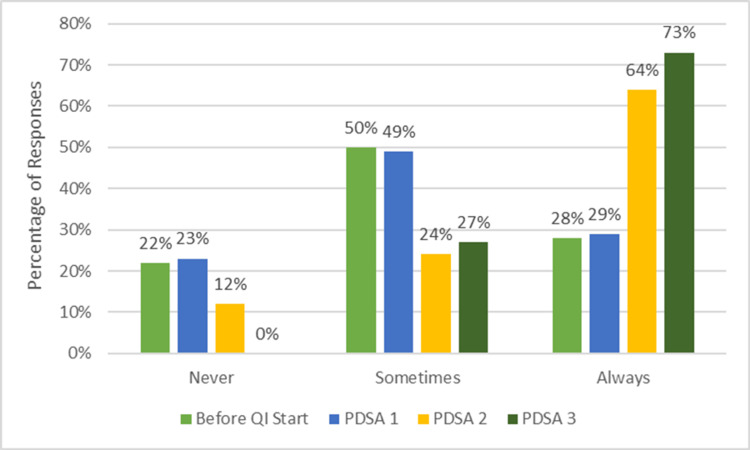
Reported frequency of notifying the on-call pediatric cardiology attending physician for after-hours echocardiogram orders.

Following the introduction of weekly email reminders and verbal awareness sessions, trivial improvement was observed. The ordering provider response rate was 35/48 responses (73%), and “Always” notifying the on-call cardiologist increased from a baseline level of 28% to only approximately 29% (Figure 3), reflecting the limitations of relying solely on educational interventions. Qualitative feedback during this cycle indicated that providers frequently forgot to notify the cardiologist in the midst of busy clinical duties, suggesting that reminders alone were insufficient to produce consistent behavior change.

Cycle 2 (December 2020-July 2021)

Educational posters were placed in workrooms and reinforced with periodic email and verbal reminders. This further improved compliance. During this period, the ordering provider response rate was 30/48 responses (63%), and “Always” notifying the on-call cardiologist rose to 64% (Figure 3). Providers reported that visible reminders in their workspaces helped prompt notification more consistently, and some described increased comfort with communication expectations. However, variability persisted, with lapses continuing during high-volume clinical shifts, underscoring the need for a more embedded, mandatory system-level solution.

Cycle 3 (August-December 2021)

The most substantial improvement occurred following the implementation of an EHR modification requiring mandatory documentation of the indication and a reminder for notification before order completion. Survey responses (30/48 responses, 63%) demonstrated a high level of compliance with the intervention (>70%). Specifically, 73% of respondents reported “Always” performing the targeted practice. The remaining respondents indicated “Sometimes,” and zero respondents selected “Never” (Figure 3). These findings highlight that, while consistent adherence was not universal, every respondent engaged in the practice at least intermittently, reflecting a significant improvement from baseline.

Sustainability

Post-cycle monitoring for up to 6 months confirmed that the high self-reported compliance achieved after the EHR intervention was maintained beyond the active QI period. Data demonstrated a sustained upward shift in communication compliance, reflecting effective integration of the intervention into routine clinical practice. Additionally, survey feedback during this phase revealed increased provider satisfaction, with many participants noting greater clarity in expectations and reduced ambiguity regarding communication responsibilities. This represented not only an immediate improvement but also a durable change that standardized workflow regardless of provider turnover or competing clinical demands.

Secondary findings

**Figure 4:**
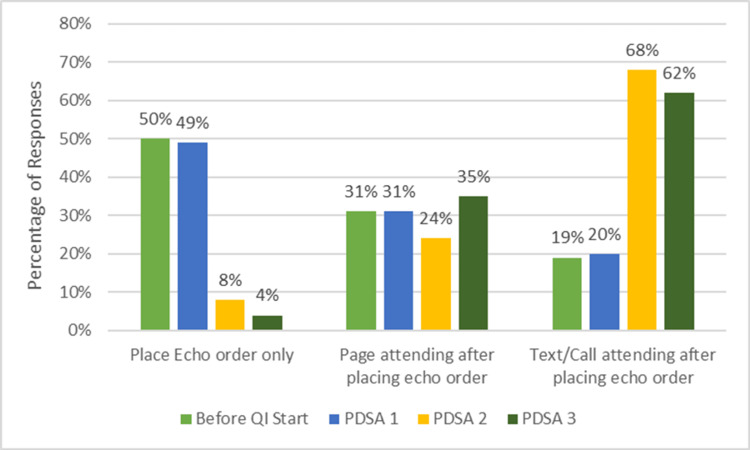
Primary methods used to notify the pediatric cardiology attending physician after placing echocardiogram orders on weekends and after hours.

**Figure 5:**
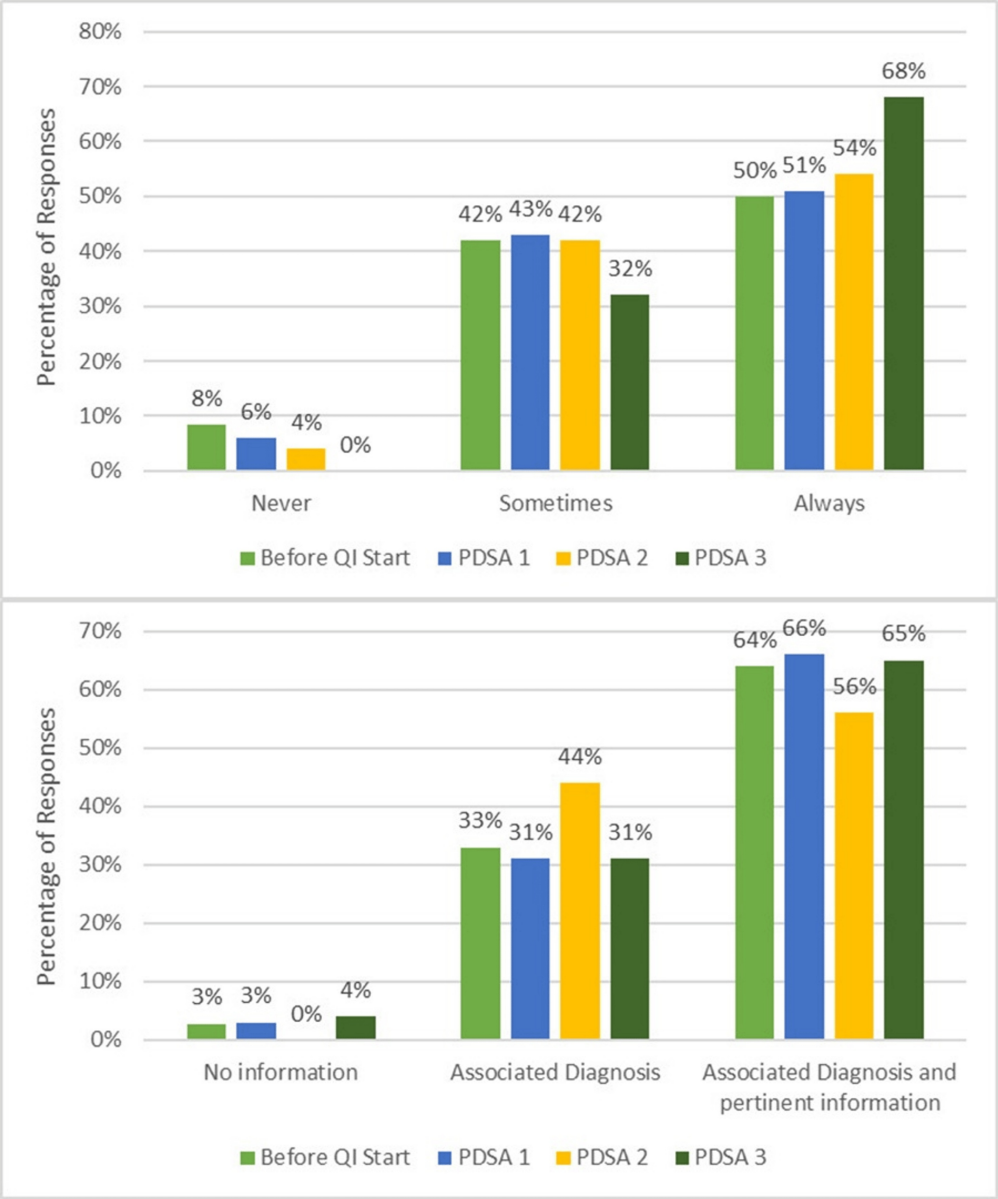
Frequency (above) and type (below) of additional information included in echocardiogram orders.

Analysis of order completeness was performed through direct review of each echocardiogram order by the pediatric cardiologists involved in the project. Their qualitative assessment demonstrated progressive improvement across cycles, with more consistent inclusion of key clinical elements, such as indication, urgency, and relevant patient condition, by Cycle 3. These findings aligned with trends observed in the self-reported surveys, which similarly reflected increased provider awareness and attention to order detail. Figures 3-5 illustrate both the increase in the frequency of inter-provider communication and the increased amount of clinical information provided. Providers highlighted that structured prompts within the EHR facilitated more thorough documentation, improving both clarity for the cardiology team and efficiency in patient management.

**Figure 6:**
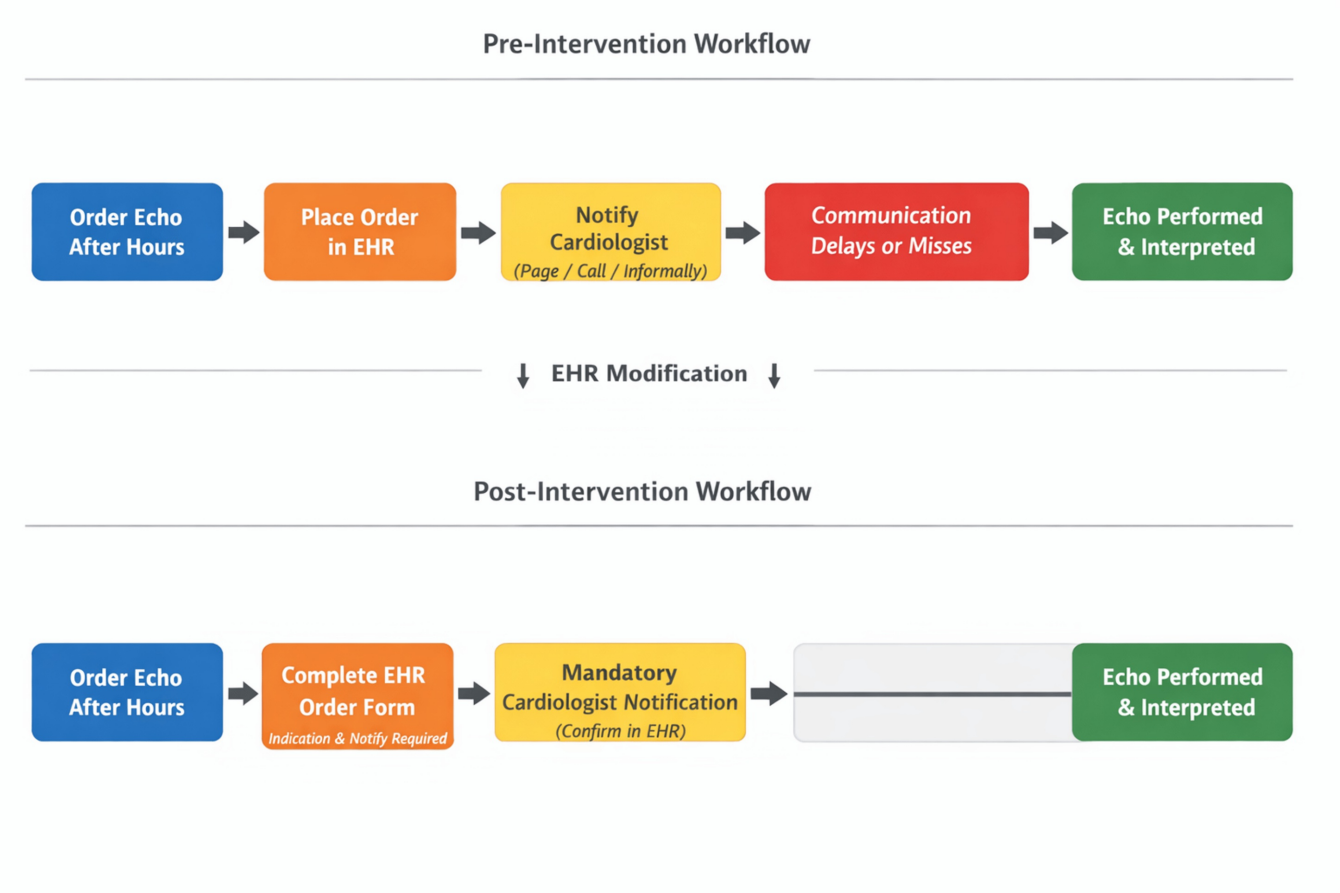
Process flow diagram showing the pre-intervention workflow, with variable cardiologist notification, and the post-intervention workflow, incorporating a mandatory EHR-based notification step to standardize communication for after-hours echocardiography orders. EHR: Electronic health record.

Overall, embedding requirements within the EHR produced the most significant and sustained improvement in communication practices. The process flow diagram in Figure 6 illustrates the workflow before and after implementation of the intervention.

## Discussion

This resident-led QI project demonstrated that structured, sequential interventions can address communication lapses in after-hours echocardiogram orders. At baseline, fewer than 30% of survey respondents reported “Always” communicating with the on-call pediatric cardiologist, underscoring a significant gap in provider practice and a potential patient safety risk. This aligns with prior observations of suboptimal baseline compliance in echocardiography practice [3-5].

Our staged approach using PDSA cycles revealed important insights. The first two cycles, which relied on educational reminders, including emails, posters, and verbal prompts, produced only incremental improvements. These findings are consistent with prior literature demonstrating that educational interventions can temporarily raise awareness but often fail to yield durable behavioral change when not supported by systems-level modification [3,6,7]. For instance, Ashraf H et al. [3] reported that decision-support tools reduced inappropriate echocardiogram ordering only when embedded into clinical workflows, rather than when offered as optional guidance.

The most significant and sustained improvement in our project occurred following the implementation of the EHR modification, where self-reported compliance exceeded our goal of 70% and remained high over the following six months. This highlights the central role of systems-level intervention in achieving lasting change [7]. By embedding notification requirements directly into the ordering process, we reduced reliance on individual memory or motivation and created a standardized workflow. These findings align with consensus recommendations emphasizing the importance of standardized documentation and structured communication in pediatric echocardiographic practice [8,9].

Improved communication in echocardiography ordering is more than an administrative outcome; it carries important implications for patient safety [2]. Delayed or missed echocardiogram orders can contribute to delayed diagnoses, inappropriate management, and increased morbidity. Prior studies have shown associations between delayed echocardiography and worse clinical outcomes in critical conditions [10]. Ensuring timely and complete provider-to-provider notification is therefore essential to minimize preventable delays in pediatric care.

Our project also highlights the value of resident-led QI initiatives. Engaging trainees as leaders not only fosters accountability and ownership but also builds a culture of continuous improvement. Prior QI reports have demonstrated the unique role of resident-driven interventions in shaping sustainable changes in institutional practice [4,5]. In our project, resident involvement in both the design and implementation phases ensured that interventions were practical and tailored to frontline workflows.

There are several limitations to our study. It was conducted at a single institution with a relatively small sample size, and certain measures relied on provider self-report, which introduces the potential for response bias. Although the absolute number of self-reported survey responses was modest, it represented more than 60% of participating providers, an overall strong response rate for survey-based research. Nonetheless, as with all survey studies, reliance on voluntary responses inherently limits generalizability and may influence the robustness of our findings. In addition, objective EHR-derived compliance data were not available for analysis; therefore, statistical process control methods could not be applied to formally assess special cause variation. Finally, while the EHR intervention was highly effective within our institution, implementation in other health systems may require adaptation to local IT infrastructure and workflow policies. Despite these limitations, the sequential approach described here offers a reproducible framework that could be applied across other pediatric and subspecialty settings facing similar communication challenges.

While the EHR modification proved to be the most effective strategy for improving communication reliability, it is important to acknowledge the potential trade-off between enhanced safety and increased documentation burden. Embedding notification requirements into the ordering workflow introduced an additional step for clinicians, which may contribute modestly to workload in already busy clinical environments. However, this intervention was intentionally designed to be brief and integrated into existing processes, requiring minimal additional time beyond standard order entry. Feedback from frontline providers suggested that, although documentation demands increased slightly, the greater clarity in expectations and reduction in downstream communication gaps ultimately improved workflow efficiency and reduced the need for follow-up clarification. These findings highlight the importance of balancing usability with reliability and suggest that future refinements should continue to prioritize streamlined, low-burden design while preserving the patient-safety gains achieved through system-level solutions.

Although post-intervention compliance is reported as exceeding 70%, it is important to interpret this figure in the context of workflow realities. Notably, there were no post-intervention responses indicating “Never” adherence; all respondents selected either “Always” or “Sometimes.” The remaining gap from 100% compliance reflects a systems-level limitation rather than a lack of provider engagement. While the EHR “hard stop” successfully ensured that providers acknowledged the requirement to notify the attending physician at the time of order entry, the actual notification process occurred outside the EHR and relied on a separate paging system. This introduced an additional step that could be delayed or occasionally missed due to competing clinical priorities, interruptions, or emergent patient care demands. These findings highlight the distinction between EHR-based process compliance and real-world execution of communication and suggest that future iterations of this intervention could benefit from integrated, automated notification or closed-loop communication tools to further improve reliability and approach universal compliance.

Overall, this project reinforces the principle that education raises awareness, but systems-level solutions can create practical change and sustainability. Embedding structured communication requirements within the EHR improved compliance with after-hours echocardiogram order notification and can serve as a model for other institutions aiming to reduce communication lapses in high-stakes diagnostic pathways.

## Conclusions

Embedding structured communication requirements within the EHR proved to be an effective strategy for improving after-hours echocardiography communication. This resident-led initiative demonstrates how systems-level solutions can enhance patient safety while fostering a culture of quality improvement.

Future efforts should focus on expanding this intervention beyond echocardiography to other diagnostic and procedural pathways where after-hours communication is critical, such as radiology, subspecialty consultations, and urgent procedural requests. Multicenter collaboration could help assess the generalizability of this approach across diverse institutional contexts and provide larger sample sizes for more robust analysis. Additionally, future studies should evaluate downstream patient outcomes, such as timeliness of diagnosis, length of stay, and clinical morbidity, to establish the direct impact of improved communication on patient care.
